# Electrochemical Sensing of Gallic Acid in Beverages Using a 3D Bio-Nanocomposite Based on Carbon Nanotubes/Spongin-Atacamite

**DOI:** 10.3390/bios13020262

**Published:** 2023-02-13

**Authors:** Sedigheh Falahi, Sepideh Falahi, Mashaalah Zarejousheghani, Hermann Ehrlich, Yvonne Joseph, Parvaneh Rahimi

**Affiliations:** 1 Institute of Electronic and Sensor Materials, Faculty of Materials Science and Materials Technology, Technische Universität Bergakademie Freiberg, 09599 Freiberg, Germany; 2 Faculty of Medicine, Kermanshah University of Medical Sciences, Kermanshah 6715847141, Iran; 3 Freiberg Center for Water Research-ZeWaF, Technische Universität Bergakademie Freiberg, 09599 Freiberg, Germany

**Keywords:** electrochemical, gallic acid, bio-nanocomposite, spongin, atacamite, carbon nanotube

## Abstract

Gallic acid (GA) is one of the most important polyphenols, being widely used in the food, cosmetic, and pharmaceutical industries due to its biological effects such as antioxidant, antibacterial, anticancer, antiviral, anti-inflammatory, and cardioprotective properties. Hence, simple, fast, and sensitive determination of GA is of particular importance. Considering the fact that GA is an electroactive compound, electrochemical sensors offer great potential for GA quantitation due to their fast response time, high sensitivity, and ease of use. A simple, fast, and sensitive GA sensor was fabricated on the basis of a high-performance bio-nanocomposite using spongin as a natural 3D polymer, atacamite, and multi-walled carbon nanotubes (MWCNTs). The developed sensor showed an excellent response toward GA oxidation with remarkable electrochemical features due to the synergistic effects of 3D porous spongin and MWCNTs, which provide a large surface area and enhance the electrocatalytic activity of atacamite. At optimal conditions by differential pulse voltammetry (DPV), a good linear relationship was obtained between peak currents and GA concentrations in a wild linear range of 500 nM to 1 mM. Subsequently, the proposed sensor was used to detect GA in red wine as well as in green and black tea, confirming its great potential as a reliable alternative to conventional methods for GA determination.

## 1. Introduction

Over the past few years, antioxidants have emerged as important ingredients in foods and beverages due to their numerous health benefits, such as anti-aging and anti-inflammatory properties [1]. In fact, they neutralize free radicals and reactive oxygen species that damage cells and cause chronic health problems and aging. Polyphenols, secondary plant compounds with over 8000 variants, are the most common antioxidants found in vegetables, fruits, grains, and beverages. Recently, they have been widely applied as natural additives instead of synthetic ones to improve food quality and promote health benefits [2,3,4]. Among them, GA (3,4,5-trihydroxybenzoic acid) and its derivatives have received significant attention due to their extraordinary biological features. They can be found in a variety of herbs, vegetables, and fruits, including apple peel, bile seeds, grapes, bananas, strawberries, lemons, pineapple, and oak bark, as well as in wine products and in green and black tea [5,6]. The anti-cancer [7], anti-diabetic [8], anti-allergic [9], anti-fungal [10], anti-bacterial [11], anti-inflammatory [12], neuroprotective [13], and cardioprotective properties [14] have made GA as one of the most widely used phenolic components in the medicine, food, and pharmaceutical industries. Considering these diverse biological and pharmaceutical effects, the determination of GA is of great importance. High-performance liquid chromatography [15], spectrophotometry [16], flow injection analysis [17], capillary electrophoresis [18], and chemiluminescence [19] are the conventional analytical techniques used to identify GA. However, despite the high sensitivity of these techniques, they require complicated equipment, specialized operators, complex sample preparation, and pre-treatment. In addition, on-site and in vivo analysis of GA is not possible with these methods. Since GA is an electroactive phenolic compound, electrochemical approaches such as cyclic voltammetry (CV), differential pulse voltammetry (DPV), amperometry, and electrochemical impedance spectroscopy (EIS) hold great potential for GA determination. Considering the advantages of this technology, including high sensitivity and selectivity, low cost, short measurement time, and simplicity, several studies have been performed to determine the GA electrochemically using different types of electrodes in various food and pharmaceutical matrices [4,20,21].

Carbon-based electrodes such as graphite [22], glassy carbon (GC) [23,24], and carbon paste electrodes (CPEs) [25,26,27,28] are generally used as working electrodes for the determination of GA. Compared to graphite and GC electrodes, CPEs seem to be the best choice due to their low cost, quick and easy fabrication and modification, fast regenerable surface by simple polishing on a paper, and low background current [29]. However, since CPEs are not sufficiently selective and sensitive to the target analyte, a variety of compounds (organic and inorganic) can be mixed with the carbon paste to improve their electrochemical performance [30]. Carbon nanostructures (such as graphene, and carbon nanotubes (CNTs)) [20,25,27], metal oxide nanoparticles (NPs) (such as CoO, ZrO_2_, and SiO_2_) [28,31,32], polymers [33], and the combination of them (Bi NPs/CNTs, polymer/CuO NPs, gold NPs/ZrO_2_ NPs) [26,34,35], as well as bio-nanocomposites (Fe_3_O_4_/chitosan nanocomposite) [36], are the most commonly used modifiers for proposed CPEs toward GA determination.

Due to the inherent advantages such as conductivity, excellent electrocatalytic activity, multiple preparation methods, and low cost, Cu-based NPs and nanocomposites have recently gained much interest as modifier candidates for the development of electrochemical sensors [37]. Nevertheless, the development of Cu-based nanocomposites with high specific surface areas and highly desirable electrocatalytic kinetics is a major challenge. This issue can be addressed by incorporating Cu-based nanostructures onto various 2D and 3D carbon nanomaterials, polymers, and biopolymers [37,38]. Especially renewable biopolymers, such as spongin, which represents the main structural proteinaceous component of sponges’ skeletons, remain to be in the focus of modern biomaterials science [39,40]. Spongin-based naturally occurring 3D scaffolds [41,42] have already been successfully used for enzyme immobilization [43,44] and carbonization [45], as well as in extreme biomimetics [46] for the development of new metal-oxide-containing composite materials [47,48,49,50]. Recently, we also reported that spongin, as a natural biopolymer with a microporous 3D structure, can react with Cu ions to form a porous 3D sensing system with excellent catalytic activity [38,51]. On the basis of the incorporation of atacamite (Cu_2_Cl(OH)_3_), as an electrocatalyst with enzyme-like activity, into the porous 3D sponge scaffold, we developed a simple and cost-effective enzyme-free glucose sensor for the determination of glucose in human blood serum samples [38]. On the other hand, CNTs and their composites have emerged as an attractive sensing material due to their high electrical conductivity, electrocatalytic effect, large surface area, and biocompatibility [52,53]. Thus, the integration of CNTs into a 3D Cu-spongin scaffold (Sp-At) creates a novel 3D sensing composite with synergistic effects and important features for catalysis.

In the present work, a simple, low-cost, environmentally friendly, and sensitive electrochemical sensor for the effective determination of GA based on a novel bio-nanocomposite of 3D porous spongin, atacamite, as a highly active catalyst and MWCNTs (denoted as Sp-At/MWCNT), was developed. The prepared 3D Sp-At/MWCNT composite was utilized for modification of CPE and applied for electrocatalytic oxidation of GA in red wine and black/green tea samples.

## 2. Materials and Methods

### 2.1. Reagents

Graphite powder, GA monohydrate (HPLC > 98%), sodium phosphate (Na_2_HPO_4_ and NaH_2_PO_4_), and paraffin oil were purchased from Sigma Aldrich. Sulfuric acid 98% and nitric acid 65% were obtained from Merck. Spongin scaffolds of Hippospongia communis marine demosponges were purchased from INTIB GmbH (Freiberg, Germany). Cupric chloride dihydrate and ammonia solution were obtained from Riedel-de Haën AG (Seelze, Germany), Th. Geyer GmbH & Co. KG (Renningen, Germany) and abcr GmbH (Karlsruhe, Germany). MWCNTs were purchased from Sigma Aldrich (Burlington, NJ, USA) with a diameter ranging from 6 to 13 nm and a length ranging from 2.5 to 20 μm. Pristine MWCNTs were then purified through acid treatment according to Wang et al. [54].

### 2.2. Apparatus and Measurements

Electrochemical measurements were performed with a PalmSens 4 electrochemical analyzer system with the software PSTrace 5.8 (PalmSens BV, Houten, the Netherlands). All experiments were carried out at room temperature in an electrochemical cell with a three-electrode platform. A saturated silver/silver chloride (Ag/AgCl) (3 M KCl solution) and a platinum wire electrode were used as the reference electrode and auxiliary electrode, respectively. Bare and modified CPEs served as the working electrode. Phosphate buffer solution (0.1 M, pH 3.0–6.0) was prepared using a mixture of the stock solutions (NaH_2_PO_4_ and Na_2_HPO_4_) and employed as an electrolyte solution for all measurements. EIS measurements were carried out in 0.1 M KCl containing 5 mM [Fe(CN)_6_]^3−/4−^ at the frequency range of 0.1 Hz–100 kHz, applied potential of 0.23 V, and an amplitude of 5 mV.

### 2.3. Preparation of the CPEs

Three-dimensional Sp-At was prepared according to our earlier reports [38,51]. It was characterized using different methods such as high-resolution transmission electron microscopy, scanning electron microscopy, X-ray photoelectron spectroscopy, and X-ray diffraction [38,51]. The bare CPE and modified CPEs were fabricated by grinding different ratios of graphite, MWCNTs, Sp-At powder, and paraffin oil as a binder in a mortar and with a grinding time of 40 min (Table 1). The components were homogenized to shape a paste, which was then pressed into a carbon paste holder with an inner diameter of 4 mm. These electrodes were then denoted as MWCNTs/CPE (MWCNTs modified CPE), Sp-At/CPE (Sp-At modified CPE), and Sp-At/MWCNTs/CPE (Sp-At- and MWCNT-modified CPE). Before each measurement, the surface of the CPEs was regenerated by smoothing with a filter paper.

### 2.4. Preparation of Sample Solutions

Black and green tea samples were obtained by a simple filtration method. A total of 3 g of dried and powered tea leaves were added to 25 mL of 80 °C boiled double distilled water for 10 min and then allowed to cool at room temperature. The extract was filtered with filter paper and diluted with 0.1 M phosphate buffer (pH 4). Prior to each measurement, it was spiked with standard GA solutions.

The red wine sample was directly diluted with 0.1 M phosphate buffer (pH 4), without any pretreatment, and spiked with standard GA solutions.

## 3. Results

### 3.1. Electrochemical Behavior of CPEs towards GA Oxidation

**Figure 1:**
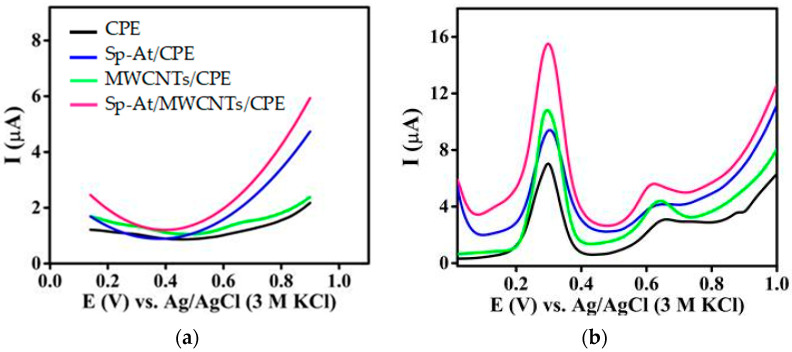
DPVs of CPE, Sp-At/CPE, MWCNTs/CPE, and Sp-At/MWCNTs/CPE in 0.1 M phosphate buffer (pH 4) at a scan rate of 0.1 V·s^−1^ in the (**a**) absence of GA and (**b**) presence of 30 μM GA.

**Scheme 1:**
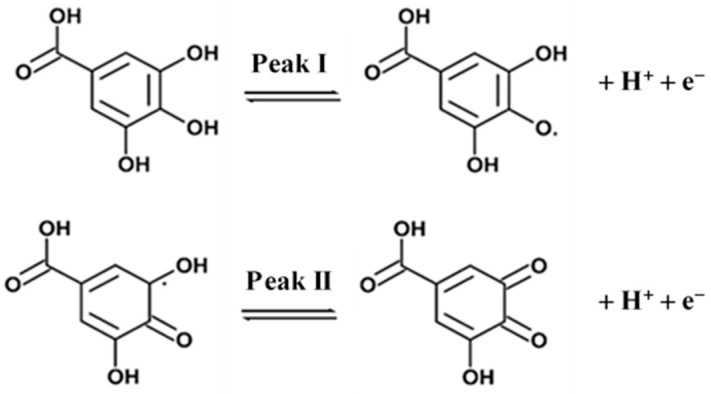
Proposed pathway for electrochemical oxidation of GA.

Figure 1 shows the DPVs of bare CPE, Sp-At/CPE, MWCNTs/CPE, and Sp-At/MWCNTs/CPE recorded in 0.1 M phosphate buffer, pH 4, in the absence and presence of 30 μM GA. In the absence of GA, no obvious oxidation peak was observed at any of the electrodes, and also the background current was very low (Figure 1a). DPVs of different CPEs in the presence of 30 µM GA revealed that the electrochemical oxidation of GA was defined by two anodic peaks (Figure 1b). Peak I (0.3 V vs. Ag/AgCl) was ascribed to the formation of the semiquinone radical as a result of the oxidation of the galloyl group, which was then oxidized to the quinone form (peak II (0.6 V vs. Ag/AgCl)) [55]. In both reactions, one proton and one electron were involved. Scheme 1 illustrates the well-known route for the oxidation of phenols, hydroquinones, and derivatives. Since the second peak was much weaker than the first one, GA oxidation was studied by focusing on the first oxidation peak [25]. As seen in Figure 1b, Sp-At/MWCNTs/CPE exhibited a clearly enhanced oxidation current in response to GA in comparison to CPE, Sp-At/CPE, and MWCNTs/CPE. In accordance with the literature [56,57], the electrochemical oxidation of GA is initiated by the oxidation of copper oxides to their higher valence states, and then the oxidized copper oxides help to oxidize GA electrocatalytically. The 3D spongin structure provides a large surface area for the formation of nano- and microcrystalline atacamite and promotes surface adsorption and electron transfer between GA and Sp-At [38]. These features, combined with the ability of MWCNTs to facilitate electron transfer kinetics, favorably contributed to the enhancement of electron transfer between GA and the electrode surface and the resulting oxidation current [27]. Hence, the Sp-At/MWCNTs/CPE was used as a working electrode for further investigations.

### 3.2. Electrochemical Characterization of CPEs

**Figure 2:**
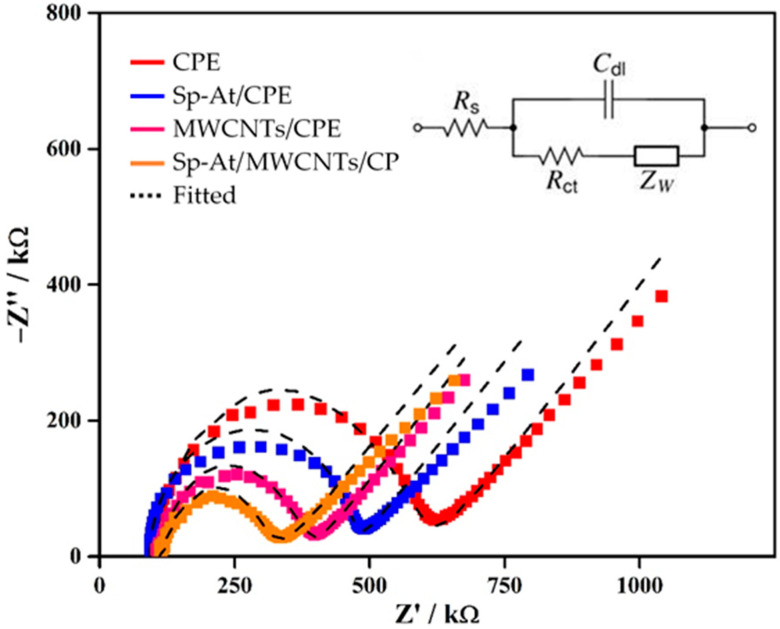
Nyquist plots of CPE, Sp-At/CPE, MWCNTs/CPE, and Sp-At/MWCNTs/CPE in a solution of 0.1 M KCl containing 5 mM [Fe(CN)_6_]^3−/4−^ at the frequency range of 0.1 Hz–100 kHz, applied potential of 0.23 V and an amplitude of 5 mV. Inset: The Randles-equivalent circuit model.

EIS, as a powerful method for characterizing the electrical properties of an electrode surface, was utilized to confirm the electrode modification. Figure 2 depicts Nyquist plots of CPE, Sp-At/CPE, MWCNTs/CPE, and Sp-At/MWCNTs/CPE in 0.1 M KCl containing 5 mM [Fe(CN)_6_]^3−/4−^. The impedance data were fitted on the basis of the Randles equivalent circuit (Figure 2, inset), where R_s_ stands for solution resistance; C_dl_ is the double-layer capacitance; and Z_w_ and R_ct_ represent Warburg resistance and charge transfer resistance, respectively. As illustrated in Figure 2, all electrodes exhibited semicircles of differing diameters in the high-frequency region, which corresponds to R_ct_ and, in the low-frequency region, a line at about 45 degrees (also known as the Warburg element), which can be attributed to the probe’s diffusion from the bulk solution to the electrode–solution interface. In comparison to the bare CPE (487 Ω), Sp-At/CPE (370 Ω), and MWCNTs/CPE (256 Ω), the Sp-At/MWCNTs/CPE (200 Ω) exhibited a smaller R_ct_ value. This indicates that both Sp-At and MWCNTs accelerate electron transfer, but the combination of 3D porous Sp-At, which offers higher electron conduction paths, and MWCNTs with a specifically large surface area exhibits the highest charge transfer and electrical conductivity.

### 3.3. Optimization of Sp-At/MWCNTs/CPE Composition

**Figure 3:**
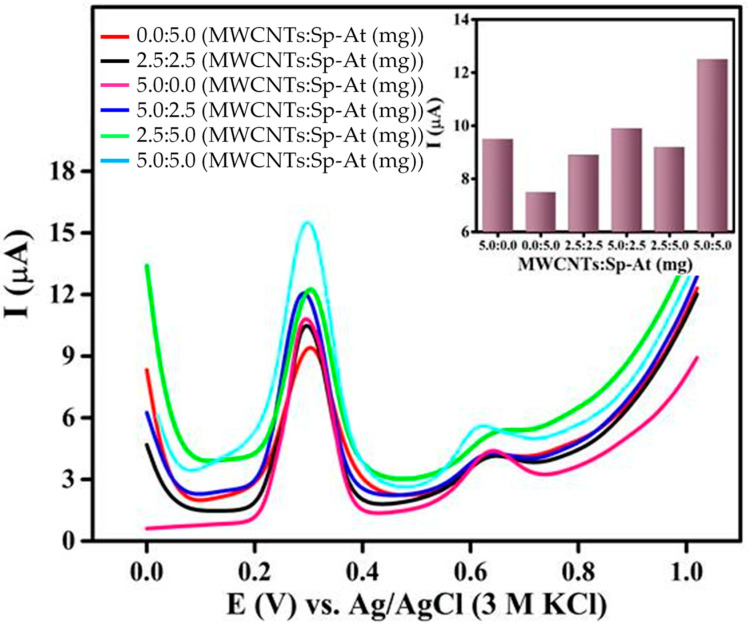
Effect of the MWCNTs/Sp-At ratio on GA oxidation (DPVs recorded in 0.1 M phosphate buffer, pH 4, containing 30 μM GA at a scan rate of 0.1 V·s^−1^). Inset: bar graphs corresponding to each electrode current response.

It is well known that the electrochemical behavior of modified carbon paste electrodes strongly depends on the mixing ratio, the way of preparing the carbon pastes, the duration of grinding, and homogenization [58,59]. In this regard, to improve the efficiency of the proposed sensor based on Sp-At/MWCNTs/CPE for detecting GA, the ratio of MWCNTs and Sp-At powder as modifiers was optimized (Table 1). Six electrodes with different ratios of MWCNTs and Sp-At powder were prepared, and the DPVs of the prepared electrodes were recorded in 0.1 M phosphate buffer, pH 4, in the presence of 30 µM GA. Figure 3 shows the responses of different electrode compositions in the similar electrochemical conditions. As can be seen, the amount of the two modifiers played an important role in the response of the sensors to GA. MWCNTs as modifiers enhanced the oxidation current of GA due to their large surface area and electrical and electrocatalytic properties. On the other hand, the 3D microporous sponges provided a larger specific surface area for atacamite as an electrocatalyst, facilitating the diffusion of GA toward atacamite and improving the electron transfer between GA and atacamite. Thus, the synergistic effects of MWCNTs and 3D Sp-At lead to a remarkable improvement in the sensing performance of Sp-At/MWCNTs/CPE. According to the obtained results, the CPE containing 10% (in a ratio of 1:1) MWCNTs and Sp-At powder showed the maximum oxidation current.

### 3.4. Influence of pH on GA Oxidation

**Figure 4:**
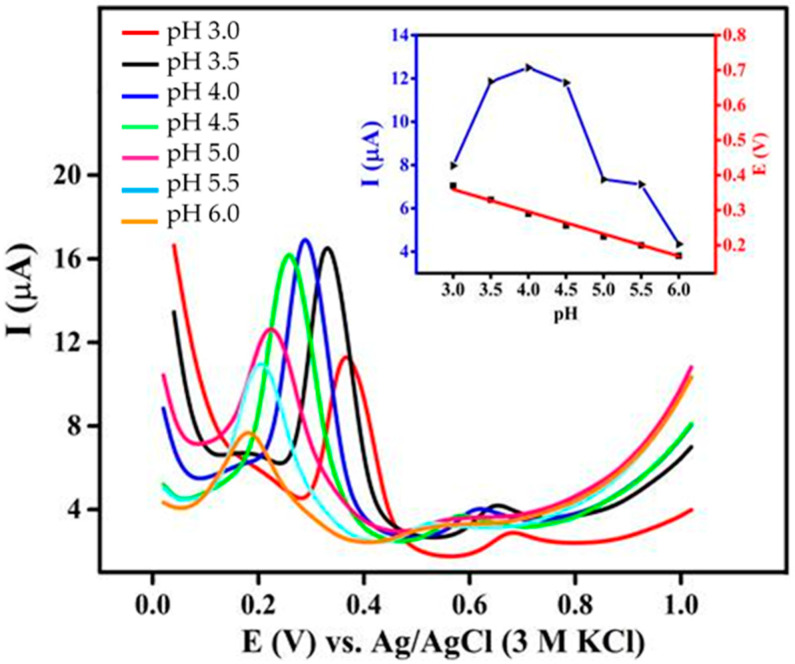
DPVs of 0.1 M phosphate buffer (pH of 3, 3.5, 4, 4.5, 5, 5.5, and 6) containing 30 μM GA at a scan rate of 0.1 V·s^−1^. Inset: effect of pH on oxidation peak current (blue) and oxidation peak potential (red).

The effect of pH on the oxidation of 30 μM GA was investigated in the pH range of 3–6 in 0.1 M phosphate buffer. It can be seen that GA oxidation was affected by the pH of phosphate buffer, which was due to the fact that GA oxidation was a protonation reaction (Figure 4). GA with a pKa of 4.4 was a weak acid and existed in the undissociated form in solution at lower pH values (pH < 4.4) and dissociated as the solution pH increased. Therefore, the maximum adsorption of GA on the surface of Sp-At/MWCNTs/CPE was expected to occur when GA was undissociated [30]. As shown in the inset of Figure 4 (blue), the anodic peak current (I_pa_) reached a maximum value at pH 4 and then decreased with increasing pH to 6. This implies that the species were gradually deprotonated. Moreover, the peak anodic potentials shifted negatively with an average shift of 30 mV when the pH was increased, confirming that the electrode process was affected by the protonation reactions, as observed by other authors [55,60]. A linear regression line was obtained for the changing of peak potential (E_p_) as a function of pH (Figure 4, inset; red) with a regression equation of E_p_ = −0.0661 pH + 0.5603 (R^2^ = 0.9929). The slope of −66 mV was in good agreement with the theoretical Nernstian systems with a mono-electron transfer step followed by single protonation [61].

### 3.5. Scan Rate Influence on the Oxidation of GA

**Figure 5:**
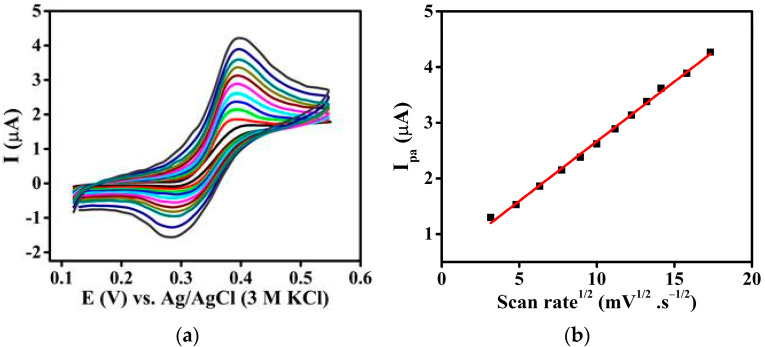
(**a**) CVs of Sp-At/MWCNTs/CPE in 0.1 M phosphate buffer (pH 4) containing 30 μM GA at different scan rates (20–300 mV·s^−1^). (**b**) The plot of the relationship between I_pa_ and the square root of the scan rate.

The GA oxidation reaction kinetics on the surface of Sp-At/MWCNTs/CPE was further evaluated by conducting CV measurements at different scan rates in 0.1 M phosphate buffer (pH 4) containing 30 μM GA solution. As can be observed, at low scan rates, a cathodic peak with very low current on the reverse sweep appeared, which indicated that the oxidized form of GA was not reduced at the surface of Sp-At/MWCNTs/CPE or maybe underwent a further chemical reaction. However, with increasing scan rate, the cathodic current increased, indicating that the oxidized form of GA was quasi-reversibly reduced before the chemical reaction [20,32]. From Figure 5a, it can be seen that the I_pa_ for GA oxidation increased as the scan rate rose from 20 to 300 mV·s^−1^. As exhibited in the Figure 5b, I_pa_ increased linearly in proportion to the square root of the scan rate with a linear regression equation of I_pa_ (μA) = 0.2147 ν^1/2^ (mV^1/2^ s^−1/2^) + 0.5186 (R^2^ = 0.9975). The R^2^ value of I_pa_ vs. the square root of the scan rate was almost equal to the theoretical value of 1.0, which was generally expected for a diffusion-controlled redox system, thus verifying that electrochemical oxidation of GA at the surface of Sp-At/MWCNTs/CPE is entirely diffusion controlled [62].

### 3.6. Quantification of GA by DPV

**Figure 6:**
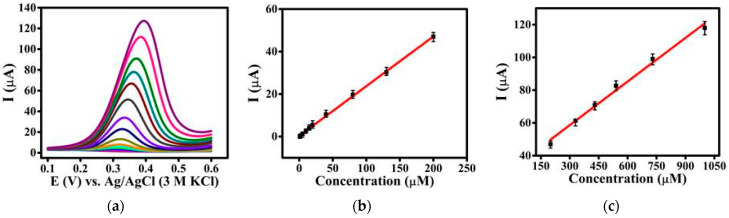
(**a**) DPV responses of Sp-At/MWCNTs/CPE with successive additions of different concentrations of GA from 500 nM to 1 mM in 0.1 M phosphate buffer (pH 4) at a scan rate of 0.1 V·s^−1^, pulse amplitude of 0.1 V, and pulse time of 0.02 s; current–GA concentration calibration curve for (**b**) low and (**c**) high concentrations of GA.

GA quantification was performed under the optimum experimental conditions (phosphate buffer (pH 4); Sp-At/MWCNTs/graphite/paraffin oil, 5:5:70:20 (W/W/W/W)). While the peak current increased by increasing the GA concentration, shift of the peak potentials towards more positive potentials was observed due to the generation of more electro-inactive oxidation species that block the electrode surface [61]. The calibration curves for the peak current versus the concentrations of GA were obtained at Sp-At/MWCNTs/CPE with two different linear parts. Figure 6b illustrates the first linear part, which was observed between 500 nM and 230 µM for lower GA concentrations with a linear regression equation as I_pa_ (µA) = 234.1 C_GA_ (mM) + 0.299 (R^2^ = 0.9991), and Figure 6c shows the second linear part for higher GA concentrations between 230 µM and 1 mM with a linear regression equation of I_pa_ (µA) = 88.8 C_GA_ (mM) + 32.024 (R^2^ = 0.9916). The sensitivity of the prepared GA sensor based on Sp-At/MWCNTs/CPE was calculated to be 18.6 μA mM^−1^ cm^−2^ at low concentrations and 7 μA mM^−1^ cm^−2^ at high concentrations of GA. The detection limit (LOD) and limit of quantitation (LOQ) were calculated from the LOD = 3 σ/m and LOQ = 10 σ/m equations, respectively (σ is the standard deviation of the signal in blank solution, and m is the slope of the calibration curve for lower GA concentrations). The LOD and LOQ values of Sp-At/MWCNTs/CPE were obtained as 5.4 nM and 17.9 nM, respectively, which were quite low in comparison to the other reported GA sensors [26,36,63]. The shift of the peak potential towards more positive potentials was due to the generation of electro-inactive oxidation species on increasing the GA concentration. These products blocked the electrode surface.

### 3.7. Quantification of GA by Amperometry

**Figure 7:**
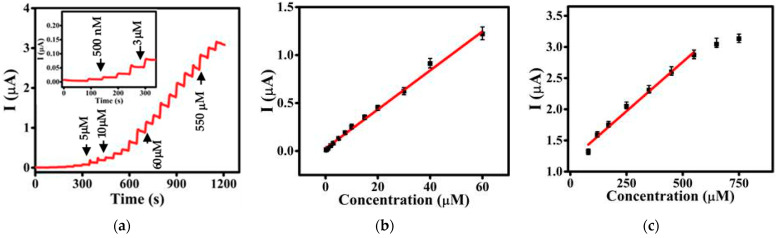
(**a**) Amperometric response of Sp-At/MWCNTs/CPE with successive additions of different concentrations of GA from 250 nM to 550 μM in 0.1 M phosphate buffer (pH 4) at 0.35 V. Inset: partial magnified view of the calibration curve for low concentrations of GA from 250 nM to 3 μM. The current-GA concentration calibration curve for (**b**) low and (**c**) high concentrations of GA.

In amperometric studies, mass transfer occurs via diffusion and convection processes, which facilitate the quantitation of analytes at lower concentrations. The amperometric results under the optimized conditions for GA oxidation at 0.35 V are presented in Figure 7a. By increasing the GA concentration, the oxidation current increased. Figure 7b,c illustrates the corresponding calibration plots for GA consisting of two different slopes. The first linear section was found between 250 nM and 60 µM with an equation of I_pa_ (µA) = 20.7 C_GA_ (mM) + 0.0238 (R^2^ = 0.9954), and the second linear part was found between 60 µM and 550 µM with an equation of I_pa_ (µA) = 3.3 C_GA_ (mM) + 1.1295 (R^2^ = 0.9841). The sensitivity of the proposed GA sensor based on Sp-At/MWCNTs/CPE was evaluated as 1.64 μA mM^−1^ cm^−2^ at low concentrations and 0.26 μA mM^−1^ cm^−2^ at high concentrations, with a detection limit of 3.6 nM. These quantitative results clearly show that the fabricated Sp-At/MWCNTs/CPE-based sensor has excellent performance for GA determination. The extraordinary electronic properties of MWCNTs promote electron transfer in electrochemical reactions, and the large surface area and good crystalline structure of the 3D Sp-At porous scaffold offer a considerable number of interaction sites for such reactions, leading to the high-performance properties of the GA sensor. Table 2 summarizes the analytical performance characteristics of the Sp-At/MWCNTs/CPE with several previously reported GA sensors.

### 3.8. Stability, Reproducibility, and Selectivity of Sp-At/MWCNTs/CPE

**Figure 8:**
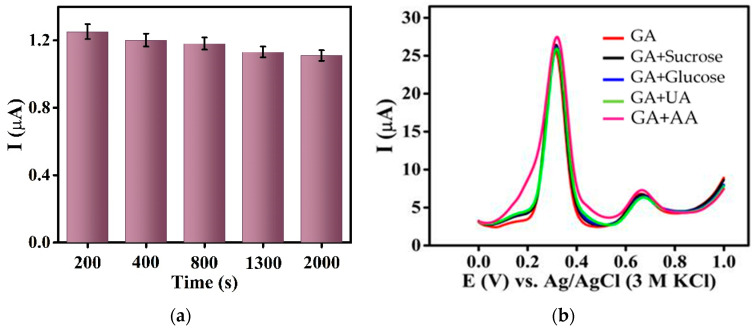
(**a**) Stability for long-term testing. (**b**) Interference test of 60 μM GA with 0.6 mM of some known interfering species.

Important factors such as selectivity, reproducibility, and stability were studied to estimate the proposed method’s applicability. To test the reproducibility, the DPV response of four Sp-At/MWCNTs/CPE fresh electrodes to 0.1 M phosphate buffer (pH 4) containing 30 μM GA was investigated (Figure S1). The relative standard deviation (RSD) of recorded anodic peak currents of four independently prepared electrodes was 4.22%, indicating that the fabricated sensors are excellent in terms of reproducibility. Similarly, in the repeatability test, eight consecutive measurements of 30 μM GA on one Sp-At/MWCNTs/CPE resulted in an RSD of 0.62%, confirming the favorable repeatability of the fabricated sensor (Figure S2). The long-term stability test was additionally assessed by DPV measurements of the Sp-At/MWCNTs/CPE in the presence of 30 μM GA. After one month, the current was still maintained at over 87% of its original value, signifying that the proposed sensor is notably stable (Figure S3). Moreover, the current response of Sp-At/MWCNTs/CPE was monitored at intervals of 2000 s after the injection of 30 μM GA. The amperometric current remained at 85% of its initial value after testing for 2000 s (Figure 8a). The specificity of the Sp-At/MWCNTs/CPE sensor was evaluated for 60 μM GA in the presence of 100-fold of possible coexistence species (sucrose, glucose, ascorbic acid (AA), and uric acid (UA)), which are usually presented along with GA [26,63]. As shown in Figure 8b, it can be found that these organic substances did not significantly impact the determination of GA. The Sp-At/MWCNTs/CPE sensor showed a relatively small peak current change, which was less than ±5%, suggesting excellent selectivity for the determination of GA. Considering that the oxidation of AA and UA occurred in the same potential window of GA, this selectivity could have been due to the structure of GA and the number of its hydroxyl groups. GA with three ionizable hydroxyl groups (compared to AA with two hydroxyl groups and uric acid without hydroxyl groups) forms both intra- (between hydroxyl groups) and intermolecular hydrogen bonds [68]. This hydrogen bonding ability contributes to GA diffusing and adsorbing more strongly than other coexisting species on the spongin scaffold (with different amino acids and consequently multiple functional groups such as SH, OH, NH_2_, and COOH) [51].

### 3.9. Determination of GA in Real Samples

Determination of GA content in black and green tea and red wine accomplished utilizing the Sp-At/MWCNTs/CPE sensor. The samples were prepared in 0.1 M phosphate buffer (pH 4) and then were spiked with standard GA solutions to obtain a GA range from 0 to 30 µM, followed by recording their corresponding DPV voltammograms (Figure S4). In all real samples (red wine, black tea, and green tea), GA was detected and quantified on the basis of the generated I_pa_ (Table 3), which is generally attributed to the total antioxidant capacity of the sample, as described [55]. More oxidation peaks were produced with increasing spiked GA concentrations in all samples. The obtained excellent recovery resulted in 95.2 to 102% (Table 3), confirming the applicability of the developed sensor for determining GA in beverages.

## 4. Conclusions

In summary, for the first time, renewable biopolymer spongin and atacamite in combination with MWCNTs (SP-At/MWCNTs) was successfully applied as a sensitive composite to fabricate a low-cost, easy, and reliable GA sensor. The SP-At/MWCNT composite enabled fast electron transfer, charge conduction, and efficient mass transport of GA on the surface of electrode. Further, the fabricated sensor based on SP-At/MWCNTs/CPE showed improved GA quantitation at a lower potential. Elevated sensitivity; acceptable selectivity among several interferences; a low detection limit; and a wide linear range with excellent stability, good repeatability, and reproducibility were realized. The developed sensor was applied successfully for determining GA in beverage samples such as black/green tea and red wine with satisfactory recovery results. The low cost, incorporation of a renewable biopolymer, ease of fabrication, and good stability make this approach attractive for the development of new sensing materials to quantify other phenolic compounds in food and beverage matrices.

## Figures and Tables

**Table 1:** Different CPE component ratios.

CPE No.	MWCNTs (%W)	Sp-At (%W)	Graphite (%W)	Paraffin Oil (%W)
1	5.0	0.0	75	20
2	0.0	5.0	75	20
3	2.5	2.5	75	20
4	5.0	2.5	72.5	20
5	2.5	5.0	72.5	20
6	5.0	5.0	70	20
7	-	-	80	20

**Table 2:** Comparison between various electroanalytical methods for the determination of GA in real samples.

Electrode	Method	Sample	LR	LOD	Ref.
Cerium-based metal organic framework on multi-walled carbon nanotubes) modified GCE (CeMOF/MWCNTs/GCE)	DPV	Tea	1.5–200 μM	0.14 μM	[64]
Poly(diphenylamine-4-sulfonic acid) modified GCE (poly(DPASA)/GCE)	CV	Honey and peanut	0.5–0.3 mM	4.35 nM	[24]
Copper-phthalocyanine-conjugated graphitic-carbon-nitride-nanosheet-modified ITO ^1^ (CuPTc/g-C_3_N_4_Ns/ITO)	DPV	Fruit juice, alcoholic beverages, and tea	10–445 nM	0.5 nM	[65]
Activated pencil lead electrode (APLE)	DPV	Black/green tea and mango juice	0.49–830 µM	0.25 µM	[66]
Fe_3_O_4_/chitosan/CPE	DPV	Green tea	0.5–300.0 μM	12.1 nM	[36]
Bismuth-nanoparticle-decorated MWCNT/CPE (Bi-MWCNT/MCPE)	Chronoamperometry	Clove and green tea	1.0–100.0 μM	0.16 µM	[26]
Reduced-graphene-oxide-modified carbon ceramic electrode (RGO)	SWV ^2^	Red wine and black/white tea	0.51–46.40 μM	86 nM	[67]
(Sp-At/MWCNTs/CPE)	Amperometry DPV	Black/green tea and red wine	0.25–550 μM 0.5–1000 µM	3.6 nM 5.4 nM	This work

^1^ Indium tin oxide. ^2^ Square wave voltammetry.

**Table 3:** Results of the analysis of GA in black/green tea and red wine.

Sample	Spiked (μM)	Detected GA (μM)	Recovery (%)	RSD (%)
Black tea	-	30.7	-	3.2
	10	40.6	98.2	2.3
	30	59.9	97	2.6
Green tea	-	31.2	-	3.6
	10	41	98	2.9
	30	62	102	3.5
Red wine	-	18.2	-	4
	10	27.8	95.2	3.3
	30	47.4	97.1	3.5

## Data Availability

Not applicable.

## Supplementary Materials

The following supporting information can be downloaded at https://www.mdpi.com/article/10.3390/bios13020262/s1. Figure S1. DPVs of Sp-At/MWCNTs/CPE in 0.1 M phosphate buffer pH 4 containing 30 μM GA at a scan rate of 0.1 V·s^−1^ (4 fresh electrodes). Figure S2. DPVs of Sp-At/MWCNTs/CPE in 0.1 M phosphate buffer pH 4 containing 30 μM GA at a scan rate of 0.1 V·s^−1^ (8 replicates). Figure S3. DPVs of Sp-At/MWCNTs/CPE in 0.1 M phosphate buffer pH 4 containing 30 μM GA at a scan rate of 0.1 V·s^−1^ (recorded within 30 days). Figure S4. DPVs of Sp-At/MWCNTs/CPE in 0.1 M phosphate buffer pH 4 containing (a) black tea sample, (b) green tea sample, and (c) red wine sample and increasing amounts of GA standard solutions. (d) Corresponding standard addition curves for determination of GA in samples.
