# Supplementary material for: Electrochemical Sensing of Gallic Acid in Beverages Using a 3D Bio-Nanocomposite Based on Carbon Nanotubes/Spongin-Atacamite
Source: Biosensors (Basel). 2023 Feb 13;13(2):262. doi: 10.3390/bios13020262 (PMC9954721; doi:10.3390/bios13020262)
Supplement: Supplementary file 1 [file biosensors-13-00262-s001.zip › biosensors-2166901-supplementary.pdf]

biosensors

Supporting Information

Electrochemical Sensing of Gallic Acid in Beverages Using a 3D Bio-Nanocomposite Based on Carbon Nanotubes/Spongin-Atacamite

Sedigheh Falahi [1], Sepideh Falahi [2], Mashaalah Zarejousheghani [3], Hermann Ehrlich [1], Yvonne Joseph [1,3] and Parvaneh Rahimi [1,3,*]

1 Institute of Electronic and Sensor Materials, Faculty of Materials Science and Materials Technology, Technische Universität Bergakademie Freiberg, 09599 Freiberg, Germany
2 Faculty of Medicine, Kermanshah University of Medical Sciences, 6715847141 Kermanshah, Iran
3 Freiberg Center for Water Research-ZeWaF, Technische Universität Bergakademie Freiberg, 09599 Freiberg, Germany
* Correspondence: parvaneh.rahimi@esm.tu-freiberg.de; Tel.: +49-3731-39-2644

3.8. Stability, Reproducibility, and Repeatability of Sp-At/MWCNTs/CPE

Important factors like selectivity, reproducibility, and repeatability were studied to estimate the proposed method's applicability. To test the reproducibility, the DPV response of four Sp-At/MWCNTs/CPE fresh electrodes to 0.1 M phosphate buffer pH 4 containing 30 µM GA was investigated (Figure S1). The relative standard deviation (RSD) of anodic peak currents was 4.22%, indicating that the prepared electrodes are highly reproducible. For the repeatability test, eight successive measurements of 30 µM GA on one Sp-At/MWCNTs/CPE yielded an RSD of 0.62%, confirming the good repeatable behavior of the fabricated sensor (Figure S2). The long-term stability test was evaluated by the DPV measurements of the Sp-At/MWCNTs/CPE in the presence of 30 µM GA. The current remained over 87% of its initial value after a month, suggesting that the proposed sensor is remarkably stable (Figure S3).

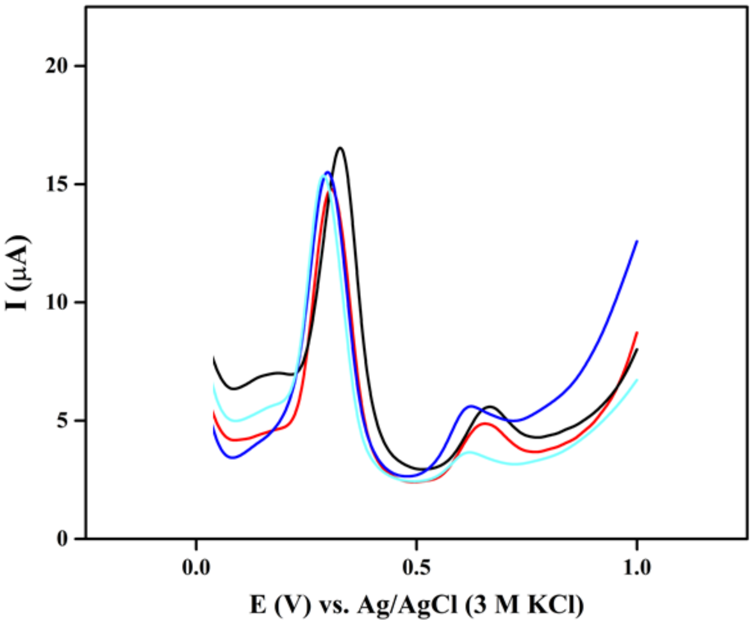

Figure S1. DPVs of Sp-At/MWCNTs/CPE in 0.1 M phosphate buffer pH 4 containing 30 µM GA at a scan rate of 0.1 V.s^{-1} (4 fresh electrodes).

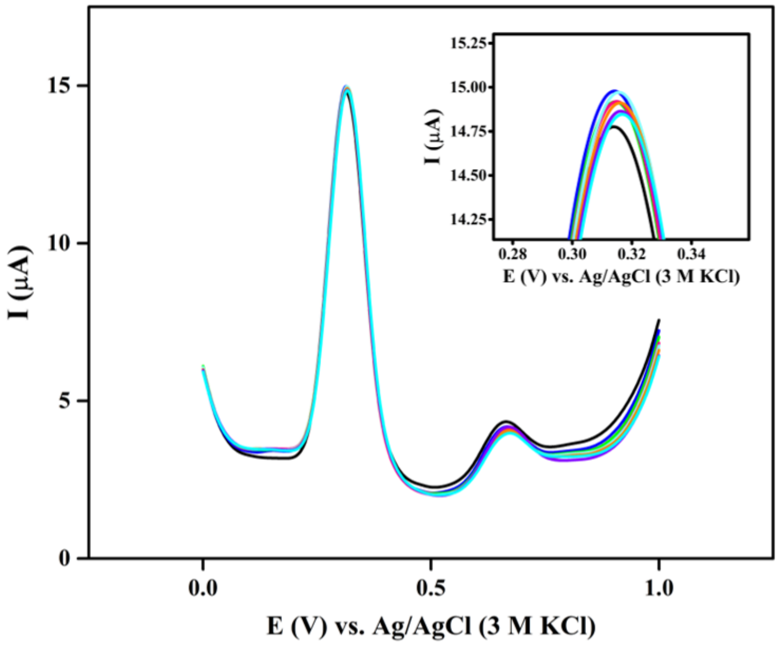

Figure S2. DPVs of Sp-At/MWCNTs/CPE in 0.1 M phosphate buffer pH 4 containing 30 μM GA at a scan rate of 0.1 V.s^{-1} (8 replicates).

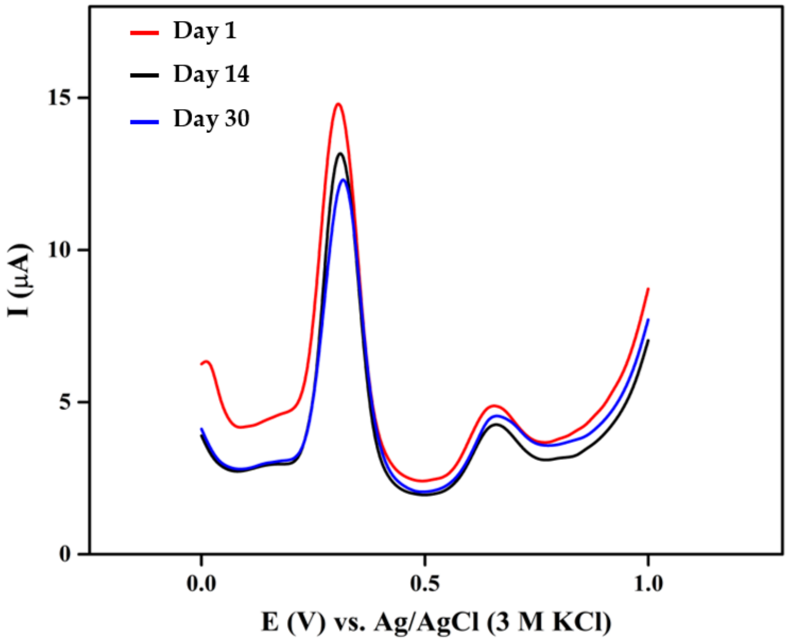

Figure S3. DPVs of Sp-At/MWCNTs/CPE in 0.1 M phosphate buffer pH 4 containing 30 μM GA at a scan rate of 0.1 V.s^{-1} (recorded within 30 days).

3.9. Determination of GA in Real Samples

Determination of GA content in black and green tea and red wine accomplished utilizing Sp-At/MWCNTs/CPE sensor. The samples were prepared in 0.1 M phosphate buffer pH 4 and then spiked with standard GA solutions to obtain a GA range from 0 to 30 μM, followed by recording their corresponding DPV voltammograms Figure S4a-c. In all real samples (red wine, black tea, and green tea), GA was detected and quantified based on the generated I_{pa}, which is generally attributed to the total antioxidant capacity of the sample, as described [55]. More oxidation peaks were produced with increasing spiked GA

concentrations in all samples. Then according to the standard addition plot as depicted in Figure S4d linear regression equations of I_{pa} (μA) = 214.3 C_{GA} (mM) + 7.2429 (R^2 = 0.9995), I_{pa} (μA) = 228.7 C_{GA} (mM) + 7.1271 (R^2 = 0.9999), and I_{pa} (μA) = 232.9 C_{GA} (mM) + 4.0286 (R^2 = 0.9928) were obtained for black tea, green tea and red wine sample standard addition curves respectively.

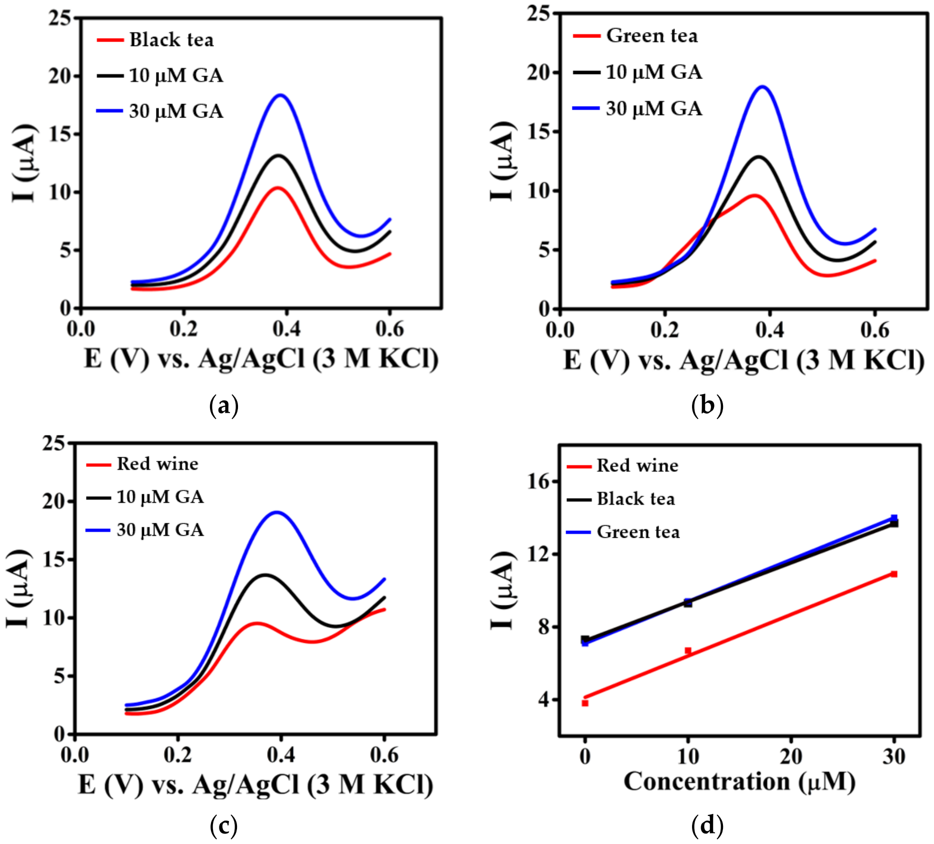

Figure S4. DPVs of Sp-At/MWCNTs/CPE in 0.1 M phosphate buffer pH 4 containing (**a**) black tea sample, (**b**) green tea sample, and (**c**) red wine sample and increasing amounts of GA standard solutions. (**d**) Corresponding standard addition curves for determination of GA in samples.
